# Neurofilaments as Biomarkers of the Efficacy of Risdiplam Treatment in Early SMA Phenotypes Diagnosed by Newborn Screening

**DOI:** 10.3390/children12091170

**Published:** 2025-09-02

**Authors:** Inmaculada Pitarch-Castellano, Nancy Carolina Ñungo-Garzón, Karolina Aragon-Gawińska, Eugenia Ibáñez-Albert, Juan F. Vázquez-Costa, Teresa Sevilla

**Affiliations:** 1 Department of Pediatrics, Hospital Universitario y Politécnico la Fe, 46026 Valencia, Spain; 2 Neuromuscular Unit, Hospital Universitario y Politécnico la Fe, Instituto de Investigación Sanitaria LaFe (IISLAFE), 46026 Valencia, Spain; 3 Centro de Investigación Biomédica en Red de Enfermedades Raras, Instituto de Salud Carlos III (CIBERER), 28029 Madrid, Spain; 4 Department of Neurology, Hospital Universitario y Politécnico la Fe, 46026 Valencia, Spain; 5 Department of Physical Medicine & Rehabilitation, Hospital Universitario y Politécnico la Fe, 46026 Valencia, Spain; 6 Department of Medicine, Universitat de València, 46010 València, Spain

**Keywords:** spinal muscular atrophy, risdiplam, newborn screening, presymptomatic SMA, 2-copies SMN2, neurofilament

## Abstract

Risdiplam is an orally administered small molecule that modifies the mRNA splicing of SMN2 for the treatment of spinal muscular atrophy (SMA). Its use is approved in presymptomatic patients diagnosed by neonatal screening with early and severe forms with two copies of SMN2, but we do not have real data on the evolution of oral treatment in this early phenotype of SMA. We present two cases treated at one month of life with a follow-up of 12 months and discuss their different evolutions and the causes of this. Familial adherence to treatment is important, as discontinuation can convert an early form of presymptomatic SMA to symptomatic. Molecular biomarkers such as plasma monitoring of neurofilament light chain (pNf-L) should be considered in the follow-up of early forms of SMA and may support the decision to change treatment in infants with SMA.

## 1. Introduction

Spinal muscular atrophy (SMA) is a neurodegenerative disease of the motor neurons in the spinal cord. A motor neuron protein (SMN) deficiency occurs, leading to progressive proximal muscle weakness that reduces the patient’s life expectancy. The estimated incidence is 1 in 6000 to 1 in 10,000 live births and the frequency of carriers is 1/40 to 1/60 [1].

It is the most common cause of infant mortality of genetic etiology. The disease is caused by homozygous mutations of the survival motor neuron 1 (SMN1) gene, while the severity of the disease is mainly related to the number of copies of the survival motor neuron 2 (SMN2) gene. The most severe forms usually have two copies and the mildest forms have four or more copies [2]. SMN2 is considered the most important phenotypic modifier, as it partially compensates for SMN1 deficiency, although we know that there are additional phenotype-modifying variables [3,4].

All three available treatments have been shown to be effective in the early or severe form of SMA. Onasemnogene Abeparvovec, Nusinersen, and Risdiplam are approved for the treatment of symptomatic or presymptomatic SMA patients with 2-copy SMN2 [5]. The most favorable therapeutic outcomes are achieved with early intervention and presymptomatic treatment, which has boosted the detection of SMA in neonatal screening (NBS) [6], compared to clinical diagnosis based on symptoms [7].

Risdiplam is an orally administered small molecule that modifies the mRNA splicing of SMN2 approved for the treatment of SMA. The clinical development of a small molecule is difficult due to the complexity of being able to determine its absorption and distribution, as well as its metabolism and elimination [8]. Risdiplam has demonstrated efficacy in pivotal trials for SMA types 1, 2, and 3 with a satisfactory safety profile (BP39056 (FIREFISH); BP39055 (SUNFISH); BN40703 (RAINBOWFISH)). The limited evidence available suggests that Risdiplam is an effective and safe drug for the treatment of SMA. In addition, the long-term clinical benefit may be determined in part by the stage of the disease at which treatment is initiated [9], as outcomes differ depending on age and the method of diagnosis (neonatal screening or clinical diagnosis based on symptoms) [7].

Real-world data in presymptomatic patients with 2-copy SMN2 treated with Risdiplam are sparse. We present the evolution of two cases diagnosed in NBS, who started treatment with Risdiplam at one month of age, being clinically asymptomatic, monitoring the activity of the disease with the detection of neurofilaments until 12 months of age.

Neurofilament proteins (Nf), both neurofilament light chain (NfL) and neurofilament heavy chain (NfH), are expressed exclusively in the cytoskeleton of neurons and have been validated as specific biomarkers of neuroaxonal injury in body fluids [10]. To assess the responses of different therapies in SMA, neurofilament measurement is beginning to be integrated into routine practice to assess people with SMA.

## 2. Case Report

Two cases of SMA were detected in NBS by quantitative PCR (qPCR) with SMN1 exon-specific probe seven and confirmed with results from SMN1 multi-ligation-dependent probe amplification (MLPA) and copies of SMN2. Both had two copies of SMN2 and neither case showed the SMN2 c.859G>C variant, which is associated with a milder phenotype [3].

The multidisciplinary team in pediatric neuromuscular diseases informed parents of the available options, the benefit/risk ratio of each drug, as well as the conditions for monitoring the treatment, following the Delphi consensus on the recommendations for the treatment of SMA in Spain (RET-SMA consensus) [11] with the three available options: Onasemnogene Abeparvovec single-dose intravenous, intrathecal Nusinersen every 4 months, and daily oral Risdiplam.

Treatment with gene therapy with Onasemnogene Abeparvovec was ruled out in both cases, with the first due to dilution of the anti-AAV9 IgG endpoint with a ratio of 1:200 with positive inferred clinical significance and the second because they were parents with habitual residence outside our country and did not guarantee the five-year follow-up required by Spanish legislation in this therapy.

The families were explained the experience of our hospital with intrathecal Nusinersen in presymptomatic patients and the lack of experience of our hospital with oral Risdiplam, but the two families opted for the oral treatment. These two patients were the first in Spain to be presymptomatic with two copies treated with oral Risdiplam.

Treatment was started at one month of age and treatments were assessed at 3, 5, 7, 9, and 12 months of age. Patients were monitored at the motor level with the Children’s Hospital of Philadelphia Infant Test of Neuromuscular Disorder score (CHOP INTEND Motor Scale (0–64)); at the neurophysiological level with compound muscle action potential (CMAP) of the right ulnar nerve amplitudes (normal > 1.5 mV) [6]; and at the analytical level with the determination of creatine phosphokinase (CPK) (normal value 30–200 U/L), Ultrasensitive Troponin T (normal value: 0.00–14.00 ng/L), and the light chain of plasma phosphorylated neurofilaments (pNf-L) (normal value < 10 pg/mL).

CASE 1. A male and the first pregnancy of healthy parents, with no family history. He was born at 38 weeks of gestation, with a birth weight of 3240 g. The diagnosis of SMA was conducted in NBS by PCR at 21 days of age, with the case confirmed by MLPA, with two SMN2 copies. On asymptomatic physical examination, the patient displayed normal muscle tone and strength, with osteotendinous reflexes present, without lingual fasciculations, with the capability of elevating the feet from the plane, and with a normal cephalic traction maneuver for the patient’s age. CHOP INTEND: 38/64 scale. Right ulnar CMAP: 0.6 mV. CPK: 225 U/L. The value of Ultrasensitive Troponin T was not determined. Neurofilaments (pNf-L): 936.71 pg/mL.

The initiation of treatment began with oral Risdiplam at 1 month of age, with increasing doses according to weight. The pNf-L values were normalized from the first moment, remaining normal throughout the follow-up. In the examination carried out in the seventh month, the motor assessment with the CHOP INTEND scale reached its maximum score, remaining at its maximum in the 9- and 12-month exams, while Ultrasensitive Troponin T as well as the right ulnar CMAP were normalized (Table 1).

The boy showed normal motor development during the first year of life, reaching the motor items successively: at 3 months stable cephalic support, at 5 months able to roll on one side, to incorporate head and trunk while lying on his stomach, and stable sitting without support, at 7 months they reached the quadriplegia position, at 9 months they were able to get up from the ground without help and reach standing with support, and at 12 months supported ambulation. Evaluation of respiratory, swallowing, and phonatory function was normal at follow-up.

CASE 2. A female and the third pregnancy of healthy parents, with two previous miscarriages, with no family history. She was born at 41 weeks of gestation, with a birth weight of 2950 g. The diagnosis of SMA was conducted in NBS by PCR at 18 days of age, with the case confirmed by MLPA, with two SMN2 copies. On asymptomatic physical examination, the patient displayed normal muscle tone and strength, with osteotendinous reflexes present, without lingual fasciculations, with the capability of elevating the feet from the plane, and with a normal cephalic traction maneuver for the patient’s age. CHOP INTEND: 38/64 scale. Right ulnar CMAP: 2.4 mV. Ultrasensitive Troponin T: 69.80 ng/L. Neurofilaments (pNf-L): 1637 pg/mL.

The initiation of treatment began with oral Risdiplam at 1 month, with increasing doses according to weight. At 6 months, during a transoceanic trip that lasted longer than expected, the girl remained without receiving the medication for 3 weeks and resumed treatment when she returned to our country.

The pNf-L values were normalized from the outset, but they increased again after 6 months, coinciding with the withdrawal of treatment for 3 weeks, without normalizing at 12 months. The same pattern emerged with the motor assessment, and the CHOP INTEND scale remained unchanged from 6 to 12 months. In this patient, the values of Ultrasensitive Troponin T have never normalized and the right ulnar CMAP was pathological from the event (Table 2).

The girl showed normal motor development until 6 months: at 3 months stable cephalic support and at 5 months able to roll on her side, to incorporate head and trunk while lying on her stomach, and stable sitting without support. At 6 months, the patient discontinued treatment for 3 weeks and in the subsequent controls (7, 9, and 12 months), she did not reach any new developmental motor items. Evaluation of respiratory, swallowing, and phonatory function was normal at follow-up.

## 3. Discussion

The implementation of neonatal screening allows us to collect information on the baseline characteristics of asymptomatic patients after a positive genetic test. According to Tizzano et al., to consider a presymptomatic patient, the manifestations of the neuromuscular phenotype (hypotonia, fasciculations, tremor, and hypo/areflexia), the respiratory phenotype (hypoxemia, hypercapnia, bell-shaped chest, and paradoxical breathing), and others such as electrophysiological studies (CMAP) and biomarkers still under study such as Nf [6] must be excluded.

Our patients did not show either neuromuscular or respiratory phenotype, but on the other hand, the values of pNf-L were elevated in accordance with the rapid loss of motor neurons that occur in early-onset forms with two copies of SMN2 and their values were high even in the absence of clinical symptoms. CPK values throughout follow-up were normal or slightly increased; they were not a good indicator for monitoring this disease and nor were the values of Ultrasensitive Troponin T.

From previous studies, we know that there is a good correlation between the determination of Nf in CSF and in peripheral blood [12], so in our clinical practice we have opted for blood measurement, which is much less invasive. We believe that biomarkers are necessary to monitor disease activity as well as response to treatment.

With the development of the anti-oligonucleotide (ASO) Nusinersen, there are studies where both neurofilament light chain (pNf-L), using ultrasensitive single molecule matrix technology Simoa® (Single Molecule Array), manufacturer by Quanterix, Billerica, MA, USA., and heavy chain (pNfH), using the ProteinSimple® (Ella immunoassay platform) manufacturer by Bio-echne, San Jose, CA, USA, as well as its correlation with Nf in CSF, have been analyzed in plasma as blood biomarkers in different types of SMA. These studies show us that the measurement of serum neurofilaments seems to differ between the subtypes of SMA in terms of usefulness as prognostic markers of the disease. pNf-L concentrations in children with early-onset SMA and two copies of SMN2 were more than 50 times higher than in individuals with late-onset SMA and >2 copies of SMN2 [10,12,13]. These data help us to understand the natural history of the disease, which is characterized by a rapid decline in functional capacities during the first 6 months of life in early-onset SMA [14]. On the other hand, neurodegeneration in late-onset SMA is not reflected in an increase in pNf-L levels, and its usefulness as a prognostic marker requires further long-term studies [13].

In our follow-up, we included the evaluation of the plasma phosphorylated neurofilament light chain pNf-L as an SMA biomarker using ultrasensitive single-molecule matrix (Simoa) technology, which can be compared with previous studies with Nusinersen [13]. This biomarker was chosen because it is routinely available in our hospital and is a good biomarker of early forms of SMA.

Both patients presented at diagnosis with clinically asymptomatic high pNf-L values. After the establishment of oral treatment with Risdiplam, its normalization indicated the effectiveness of the treatment in both cases. In CASE 1, the pNf-L values were normal throughout follow-up, but in CASE 2 the patient became symptomatic after a discontinuation of treatment for 3 weeks at the age of 6 months, with an increase in pNf-L, plateauing on the CHOP INTEND scale, and a decrease in the right ulnar CMAP.

We have recommendations based on the use of pharmacokinetics to restore Nusinersen levels in CSF with treatment interruptions, the regimen of which depends on the interval since the last maintenance dose was administered [15]. In view of the serious consequences, it would be necessary to assess the action in cases of temporary interruption of Risdiplam. The dose was restarted for weight according to the dosage guidelines of the drug, as no indication was available.

Risdiplam reaches a steady state more quickly than Nusinersen (1 week for Risdiplam vs. 2 months for Nusinersen). A different administration scheme of Nusinersen is currently underway in a phase 2/3 clinical trial (clinicaltrials.gov identifier: NCT04089566) that may result in faster achievement of steady-state levels [7].

Nusinersen reaches a maximum concentration between 1.7 and 6 h after administration, with a half-life of 135 to 177 days in CSF [16]. On the other hand, the concentration of Risdiplam depends on the weight and age of the patient according to pivotal clinical trials (BP39056 (FIREFISH); BP39055 (SUNFISH); BN40703 (RAINBOWFISH)); according to the proposed dosage, patients (with a body weight > 50 kg or an age < 1.3 years or an age > 13 years) appear to have a lower exposure, while other groups of patients have a higher exposure. However, the dosing regimen studied in pivotal clinical trials (which resulted in a lower or higher end of exposure) showed no relevant differences in terms of efficacy or safety outcomes. Risdiplam has a half-life of about 50 h [17].

Our findings support the current strategies for detecting SMA in NBS; it is also of great importance to have an early treatment protocol, especially in countries with legal or access restrictions.

In the future, the widespread detection of SMA in NBS will allow early treatment of patients with either SMN2-modifying drugs (Nusinersen or Risdiplam) or gene therapy (Onasemnogene Abeparvovec) [18]. We currently have information on the treatment of presymptomatic patients with two copies of SMN2 from clinical trials of the different drugs. Results are available with Nusinersen at 5 years with the NURTURE trial, where 13 of the 15 participants achieved ambulation (87%) [19]. Results are available with Onasemnogene Abeparvovec at 2 years from the SPRINT trial, where 9 of the 14 participants achieved ambulation (64%) [20]. And, with Risdiplam, 2-year data are available with the RAINBOWFISH trial, where 3 of the 5 participants achieved ambulation (60%) [21].

Evidence of the efficacy of treatments in SMA exists and the current treatments have been described as providing significant clinical improvements, but we know that there is intra- and inter-individual variability in motor development, so molecular biomarkers such as pNf-L should be taken into account in the follow-up of early forms of SMA and may support the decision to switch therapies in infants with SMA. In addition, the choice of drug should be seriously considered, considering the family’s adherence to treatment, since a 3-week discontinuation of treatment with Risdiplam can make an early form of presymptomatic SMA symptomatic.

However, we are aware that more cases of early forms of presymptomatic SMA treated in real life and followed with biomarkers are needed to draw conclusions about the efficacy of the different drugs in the long term. What is clear from the experience of our cases is that early detection in NBS and the initiation of treatment in the presymptomatic stage can modify the evolution of the disease.

## Figures and Tables

**Table 1:** Follow-up of Case 1.

Child (Boy)	1 Month	3 Months	5 Months	7 Months	9 Months	12 Months
CHOP INTEND (64/64)	38	48	59	64	64	64
CPK Creatine phosphokinase (30–200 U/L)	225	425	338	309	212	151
pNf-L Plasma phosphorylated neurofilament light chain (<10 pg/mL)	936.71	10.74	11.58	14.51	8.96	8.54
Ultrasensitive Troponina T (0.00–14.00 ng/L)	---	46.00	23.50	12.30	9.43	8.99
Right ulnar CMAP Peak amplitude (>1.5 Mv)	0.6	0.8	---	5.7	5.78	5.8

**Table 2:** Follow-up of Case 2.

Child (Girl)	1 Month	3 Months	5 Months	7 Months	9 Months	12 Months
CHOP INTEND (64/64)	38	48	56	56	56	56
CPK Creatine phosphokinase (30–200 U/L)	99	156	211	169	119	155
pNf-L Plasma phosphorylated neurofilament light chain (<10 pg/mL)	1.637	13.73	9.69	214.84	152.48	49.51
Ultrasensitive Troponina T (0.00–14.00 ng/L)	69.80	109.00	49.60	41.70	36.10	32.40
Right ulnar CMAP Peak amplitude (>1.5 Mv)	2.4	2.5	---	1.9	1.1	2.2

Note: At 6 months, 3 weeks of treatment were discontinued.

## Data Availability

The data that support the findings of this study are available on request from the corresponding author. The data is not publicly available due to privacy or ethical restrictions.
